# Ca^2+^‐Activated Cl^−^ Channels: Do Bestrophins and TMEM16A Interact?

**DOI:** 10.1111/apha.70232

**Published:** 2026-05-12

**Authors:** Christian Aalkjær, Vibeke Secher Dam, Holger Nilsson, Donna B. Boedtkjer, Vladimir Matchkov

**Affiliations:** ^1^ Department of Biomedicine Aarhus University Aarhus Denmark; ^2^ Department of Clinical Medicine Aarhus University Aarhus Denmark; ^3^ Department of Physiology Institute of Neuroscience and Physiology, The Sahlgrenska Academy, University of Gothenburg Gothenburg Sweden; ^4^ Aarhus Institute of Advanced Studies Aarhus University Aarhus Denmark

## Abstract

**Aim:**

Ca^2+^‐activated Cl– conductances are present in many cell types and are important for regulating membrane potential as well as other cellular functions. TMEM16A is widely accepted as the principal molecular basis for Ca^2+^‐activated Cl– conductances, but also members of the bestrophin family may be important for some Ca^2+^‐activated Cl– conductances.

**Methods:**

In this review, we discuss the possibility that members of the two families, bestrophins and TMEM16A, may interact.

**Results:**

In the cardiovascular system, the evidence is strongest. Here (1) TMEM16A and bestrophin‐3 are closely located in the membrane, (2) TMEM16A may regulate the expression of bestrophin‐3, and (3) a cGMP dependent Ca^2+^‐activated Cl– conductance is dependent on both TMEM16A and bestrophin‐3. Outside the cardiovascular system, both TMEM16A and bestrophins are implicated in the regulation of cellular Ca^2+^ independently of membrane potential, and both proteins are importantly associated with inflammation and pain transmission. However, it seems that little testing of a direct interaction of TMEM16A and bestrophins has been published.

**Conclusion:**

We conclude that there is evidence for direct interaction between TMEM16A and bestrophins in the cardiovascular system, and it will be important to determine whether similar interactions extend to other tissues.

A chloride conductance that depends on calcium for activation is termed a Ca^2+^‐activated Cl^−^ current. The conventional Ca^2+^‐activated Cl^−^ current is characterized by time‐ and voltage‐dependence (Figure 1). This conductance is present in a wide range of cell types across species. Early candidates in the extensive search for the gene product encoding the protein mediating this conductance included members of the bestrophin family. Notably, bestrophin‐2 was shown, via biotinylation assays, to localize to the plasma membrane, and amino acid substitutions were found to alter ion selectivity—evidence supporting its potential role as a plasma membrane ion channel [3].

**FIGURE 1:**
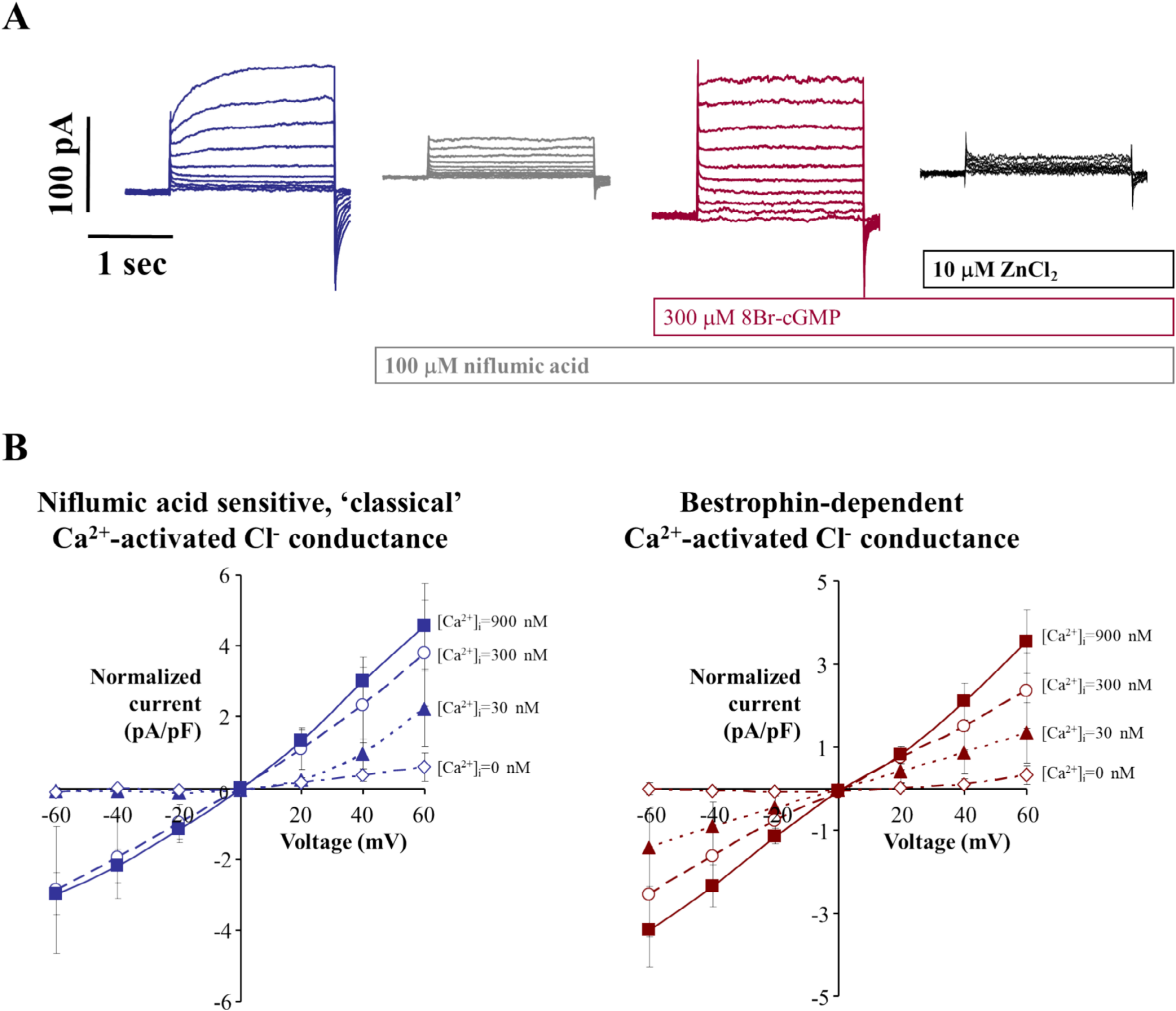
Different time‐ and voltage‐dependence of the Ca^2+^‐activated Cl^−^ conductances in vascular smooth muscle cells. (A) The Ca^2+^‐activated Cl^−^ current was activated in isolated smooth muscle cells from rat mesenteric arteries by 900 nM free Ca^2+^ in the patch pipette and studied with voltage‐steps between −90 and +90 mV with 20 mV increments. This current had a characteristic time‐ and voltage‐dependence, inhibited with niflumic acid an unspecific inhibitor of ‘classical’ Ca^2+^‐activated Cl^–^ current. In the presence of niflumic acid, cGMP activated another time‐independent Ca^2+^‐activated Cl^–^ current that showed smaller voltage‐dependence. This second current was inhibited with micromolar ZnCl_2_ concentrations. Modified from Matchkov et al. [1] under Creative Commons license, CC BY‐NC‐SA 4.0. (B) Both Cl^−^ conductances are Ca^2+^‐dependent, though the ‘classical’ conductance has strong outward rectification, especially at low intracellular Ca^2+^ concentrations. The cGMP‐dependent, ZnCl_2_‐sensitive conductance depends on bestrophin‐3 expression. Recordings are made in cultured smooth muscle cells, A7r5 with 900 nM free Ca^2+^ in the pipette. Modified from Matchkov et al. [2] in accordance with Copyright Agreement.

After some time, however, questions started to accumulate as to whether bestrophins were the molecular correlate to the previously characterized Ca^2+^‐activated Cl^−^ channels. The original description of bestrophins was connected to the finding that mutation in its gene correlated with the appearance of vitelliform macular degeneration (Best's disease) [4, 5], an eye disease affecting retinal pigment epithelium cells. This disease is characterized by specific changes (loss of the light peak) in the electrooculogram (EOG) that correspond to the loss of a Cl^−^ current [6]. Although many mutations in the bestrophin gene (VMD2) correlate with the characteristic EOG changes in Best's disease, this is not universally the case. In some mutations, typical EOG changes are absent or occur later than the onset of macular dystrophy (see Strauss [7] for references). This raised doubts as to whether the Cl^−^ channel dysfunction of bestrophin‐1 is essential for Best's disease. Unfortunately, only a limited number of the more than 300 known mutations of the human bestrophin‐1 gene [8, 9, 10, 11, 12, 13] have been characterized in terms of their impact on the channel's Cl^−^ current.

Another uncertainty of the role of bestrophins stems from analysis of their subcellular localization. In many cases, most of the bestrophin molecules have been shown to reside intracellularly [14, 15, 16], and not in the cell membrane, which is difficult to reconcile with a primary role in plasmalemmal ion transport. Speculations have thus arisen that bestrophins may be intracellular channels or that they may be regulators of other molecules that constitute the true Cl^−^ channel pore.

Another obstacle to establishing bestrophin as the molecular correlate to the known Ca^2+^‐activated Cl^−^ channel was the current characteristics: the time‐independent current of bestrophins (Figure 1) did not fit the kinetics of the known Ca^2+^‐activated Cl^−^ currents (see Hartzell et al. [17]).

The identification of TMEM16A (anoctamin 1, ANO1) a few years later provided a molecule with biophysical properties that were a better fit to those of the described Ca^2+^‐activated Cl^−^ conductances [18, 19, 20]. This led to a rapid shift of focus in research, and TMEM16A fulfilled these expectations well.

It is now established that bestrophins and TMEM16A are often co‐expressed in the same cells and can contribute to similar physiological functions. In this review, we will first discuss the evidence for direct interaction between bestrophins and TMEM16A, mainly based on findings in vascular smooth muscle cells. In the last section of the review, we will exemplify where bestrophins and TMEM16A are important for the same physiological functions in different organ systems. The conclusion of the review is that it may be worth providing a more in‐depth assessment of the interaction between TMEM16A and bestrophins.

## 1 Evidence for Direct Interaction Between Bestrophins and TMEM16A

Given that bestrophins and TMEM16A can potentially form Cl^−^ channels, often expressed in the same cells and important for the same function, it is surprising that there are only a few studies that directly address their interaction. In this section, we will review these studies.

In vascular smooth muscle cells, Cl^−^ is actively transported into the cell by means of the Na, K, Cl‐cotransporter1 and the Cl/HCO_3_‐exchanger, which elevates intracellular Cl^−^ to levels that maintain a driving force for Cl^−^ efflux upon the chloride channel opening. Chloride channels are therefore important for the membrane potential, and with a Cl^−^ equilibrium potential of approximately −30 mV, Cl^−^ efflux typically depolarizes the cell. Vascular smooth muscle cells possess several Cl^−^ conductances (see e.g., Matchkov et al. [21]).

Of relevance for this review, vascular smooth muscle cells have two distinct Ca^2+^‐activated Cl^−^ conductances. The one conductance is the conventional conductance with voltage‐ and time‐dependence [22, 23, 24, 25, 26]. Another conductance is strictly dependent on cGMP via a PKG‐mediated mechanism [1, 27], has no voltage‐ and time‐dependence, is inhibited by 10 μM Zn^2+^ (Figure 1) and is relatively insensitive to conventional chloride channel blockers, such as niflumic acid, IAA‐94 and DIDS [1, 25, 27, 28, 29]. The cGMP‐dependent Cl^−^ current is dependent on calmodulin, but independent of calmodulin‐dependent protein kinase II [29]. It is noteworthy that cGMP—typically associated with hyperpolarization and reduced intracellular Ca^2+^ in vascular smooth muscle—activates a depolarizing Cl^−^ conductance. The two Ca^2+^‐activated Cl^−^ conductances are present in smooth muscle cells from all vascular beds investigated with the exception of the pulmonary artery smooth muscle cells where only the classical conductance is present [30]. However, the relative magnitudes of these two Cl^−^ conductances vary substantially between smooth muscle cells from different vascular beds [30].

**FIGURE 2:**
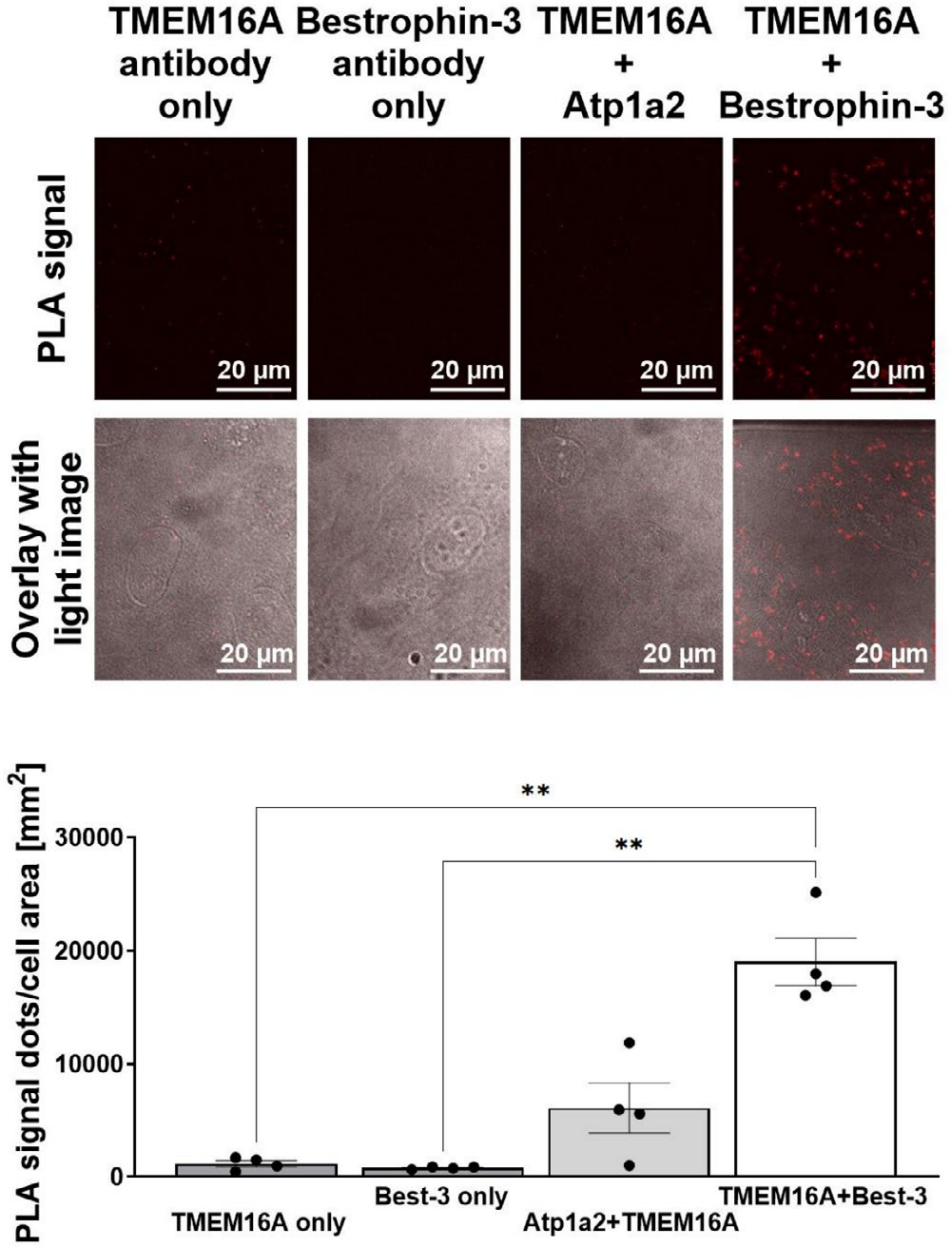
Proximal ligation assay (PLA) suggests colocalization of bestrophin‐3 (Best‐3) and TMEM16A. (A) Representative fluorescent images (excitation 554/emission 576; LSM‐5 Pascal Exciter, Zeiss, Germany) and their overlay with widefield images of A7r5 cells showing the PLA experiment results where cells were treated with either TMEM16A antibody only (ab53213, Abcam, UK), or Best‐3 antibody only (BSt‐301AP, FabGennix, USA), or by a combination of anti‐TMEM16A antibody and antibody against Atp1a2 (AB9094, Millipore, USA), or by a combination of TMEM16A and Best‐3 antibodies. Fixed A7r5 cells on coverslips were incubated with primary antibody and PLA was carried out using Duolink in situ kit (Olink bioscience, Sigma Aldrich, Denmark). (B) Images were analyzed for signal spots with ImageJ (National Institutes of Health, USA) and presented per cell area. Signal was significantly higher when applying anti‐BEST‐3 and anti‐TMEM16A antibodies together in comparison with other combinations. Averaged results of 4 independent experiments. **p < 0.01 compared using one‐way ANOVA followed with Turkey correction for multiple comparisons.

**FIGURE 3:**
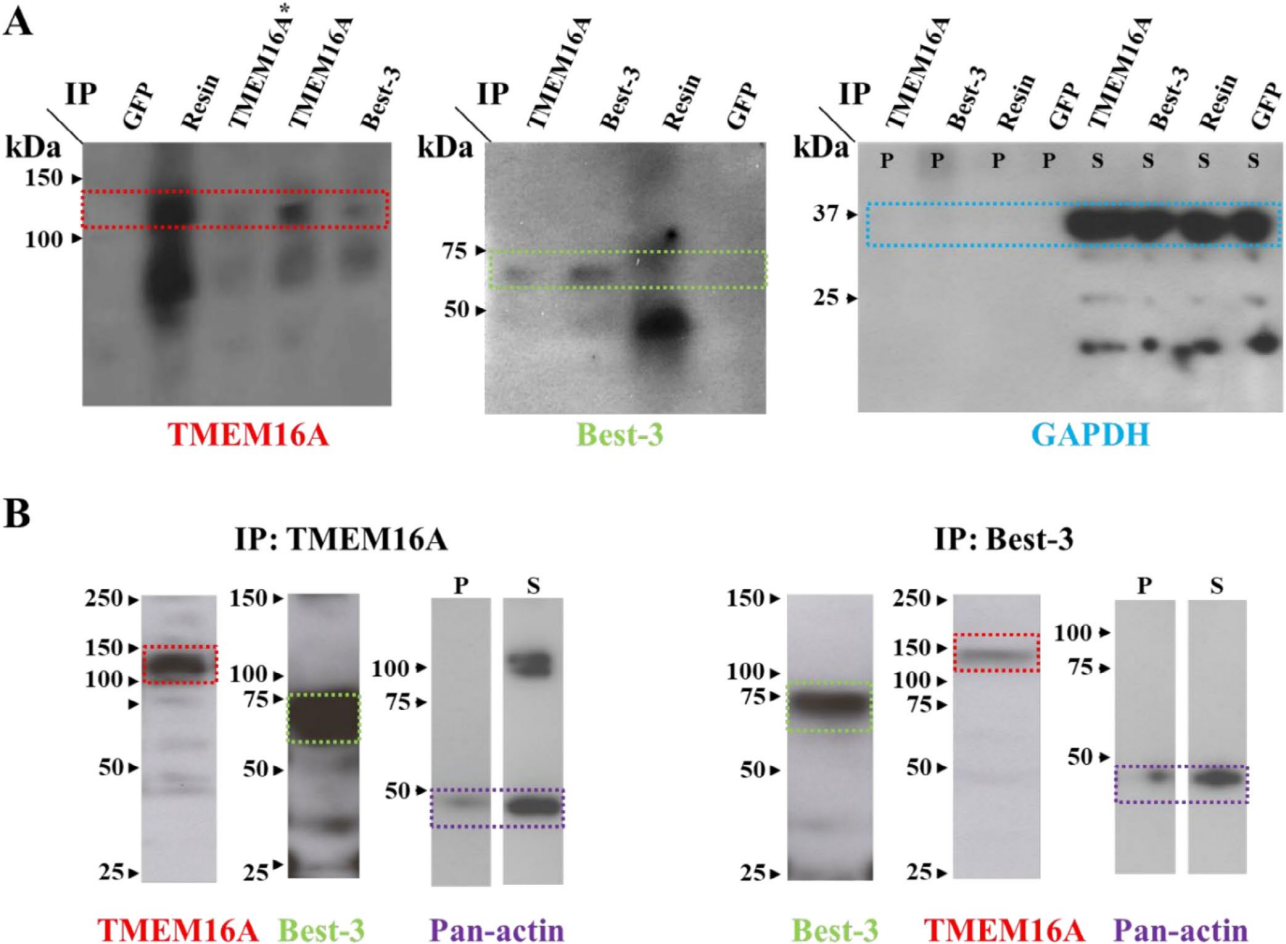
Co‐immunoprecipitation of bestrophin‐3 (Best‐3) and TMEM16A suggests physical interaction between these proteins in smooth muscle cells. (A) Lysates from cultured smooth muscle cells, A7r5 were precipitated (IP) with antibodies against either TMEM16A (ab53213, Abcam, UK or MABC36, Millipore, USA, which is labeled with *) or Best‐3 (Bst‐301AP, FabGennix, USA). Precipitate with antibody against GFP (ab290, Abcam, UK) or resin without any antibody serving as controls. Both TMEM16A and Best‐3 in the precipitates (P) were then detected with immunoblots using matching secondary antibodies (Millipore, US) and the ECL chemiluminescence system (Amersham; GE Healthcare). Immunoblot with “housekeeping” GAPDH antibody (CST‐2118, Cell Signaling Technology, USA) detected GAPDH in supernatants (S) of all samples, but not in the precipitates. Precipitates with TMEM16A antibody contained Best‐3 protein, and vice versa. (B) TMEM16A (left panel) and Best‐3 (right panel) were precipitated in rat mesenteric small artery lysate as indicated. Both TMEM16A and Best‐3 were detected on immunoblots of both lysates. Anti‐pan‐Actin antibody (CST‐4968, Cell Signaling Technology, USA) was used as a control and immunoblotted on precipitate (P) and supernatant (S).

**FIGURE 4:**
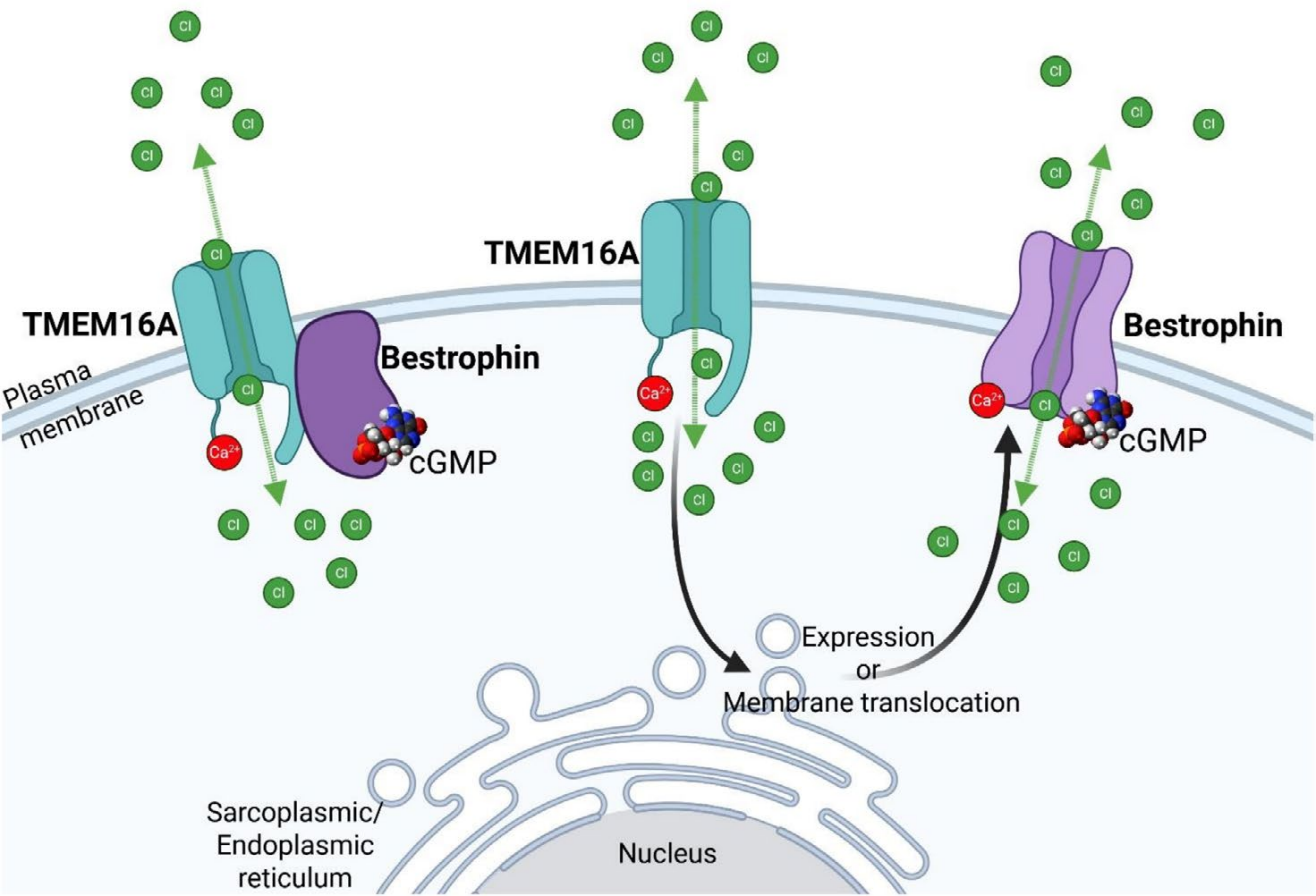
Complexity of functional interaction between TMEM16A and bestrophin proteins in forming Ca^2+^‐activated Cl^−^ conductance in the cell membrane. The functional interaction between TMEM16A and bestrophin is possible in the form of colocalization, where bestrophin acts as an accessory that modifies the gating properties of the TMEM16A Cl^−^ channel, or via TMEM16A‐dependent expression or membrane abundance of a bestrophin‐formed Cl^−^ channel.

Knockdown of TMEM16A inhibits both Ca^2+^‐activated Cl^−^ conductance types [31]. In contrast, knockdown of bestrophin‐3 eliminates only the cGMP‐dependent current induced by high Ca^2+^ in the patch pipette, while the classical current is unaffected [2]. This is confirmed by the finding that an anti‐bestrophin‐3 antibody inhibits the cGMP‐dependent current, but not the classical current in single channel recordings [28]. Additional support for bestrophin‐3 being essential for the cGMP‐dependent current is the finding that the pulmonary artery smooth muscle cells, which lack the cGMP‐dependent current, have no bestrophin‐3 at the transcript and the protein level [2]. The reliance of the cGMP‐dependent current on the presence of both bestrophin and TMEM16A implies that these two molecules interact in some way. One possibility is that sarcoplasmic‐reticulum‐located bestrophin‐3 supports release of Ca^2+^ from intracellular stores. Although this cannot be excluded, the finding that a bestrophin‐3 antibody inhibits the single channel cGMP‐dependent Cl^−^ current [28], an experimental condition where there is no sarcoplasmic reticulum and intracellular Ca^2+^ release, demonstrates that bestrophins have a direct effect in the sarcolemma. This conclusion is further supported by the finding that increase of Ca^2+^ influx induced with the Ca^2+^ ionophore ionomycyin or the L‐type Ca^2+^ channel agonist BayK 8644 can elicit the Cl^−^ current [1], although it can also be elicited by caffeine‐induced Ca^2+^ release from the sarcoplasmic reticulum [1]. Interestingly, in canine and human cardiomyocytes bestrophin‐3 has much stronger co‐localization with the L‐type Ca^2+^ channel than with the ryanodine receptor [32], consistent with bestrophin‐3 being in the sarcolemma of cardiomyocytes. A second possibility is that bestrophin is a subunit to TMEM16A: support for this possibility comes from initial colocalization evidence obtained in our laboratory, where PLA experiments (Figure 2) and a single immunoprecipitation experiment (Figure 3) strongly suggest proximity between TMEM16A and bestrophin‐3 in smooth muscle cells. Importantly, colocalization of bestrophin‐3 and TMEM16A has also been reported in canine ventricular cardiomyocytes [32], and it will be of interest to find out how common colocalization of bestrophin and TMEM16A is in other cell types. A third possibility is that the current is mediated by bestrophin‐3, and TMEM16A regulates the expression of bestrophin‐3. This possibility is demonstrated by the observation that downregulation of TMEM16A leads to downregulation of bestrophin‐3 mRNA (and also to downregulation of bestrophin‐1 and ‐2) in vascular smooth muscle cells [31]. It remains unclear whether the dependence of the cGMP‐dependent current on both proteins reflects: (1) bestrophin‐3 functioning as a subunit of TMEM16A, (2) TMEM16A regulating bestrophin expression, or (3) a combination of both mechanisms (Figure 4). It is interesting that TMEM16A may also be regulating the expression of other important molecules in smooth muscle cells [33].

In conclusion, there is strong evidence for a direct interaction between bestrophins and TMEM16A in vascular smooth muscle cells (1) at the regulatory level, (2) in the form of colocalization, and (3) with respect to the cGMP‐dependent Cl^−^ conductance (Figure 4). This interaction has consequences for vascular function. The oscillation of vascular tone, that is, vasomotion, which is a common phenomenon in most vascular beds is dependent on the Ca^2+^‐activated cGMP‐dependent Cl^−^ conductance and is inhibited with bestrophin‐3 knockdown [34] and with TMEM16A knockdown [31]. This demonstrates that also at the tissue level an interaction between bestrophin‐3 and TMEM16A is evident.

Although direct interaction between bestrophin and TMEM16A has not been convincingly demonstrated beyond the cardiovascular system, there is much evidence that bestrophins and TMEM16A are important for the same functions in cells and organs. In the next section we will exemplify this.

## 2 Role for Bestrophin and TMEM16A in the Control of Cell Ca^2+^

### 2.1 L‐Type Ca^2+^ Channels

Bestrophins and TMEM16A may affect the cell membrane potential via their function as Cl^−^ channels and consequently intracellular Ca^2+^. However, there is also evidence that they may control intracellular Ca^2+^ via other types of interactions with Ca^2+^ channels. For bestrophin‐1 there is strong evidence for an interaction with the L‐type Ca^2+^ channel (Ca_v_1.3) in retinal pigment epithelial cells [35, 36]. Accordingly, the Ca^2+^‐channel has crucial importance for the light peak in the EOG, which is bestrophin‐1‐dependent [37, 38]. Expression of bestrophin‐1 in retinal pigment cells alters the characteristics of L‐type Ca^2+^ channel activity [39], including a shift of voltage‐dependent activation to more negative values and faster activation kinetics. In other experiments the intracellular Ca^2+^ increase following ATP stimulation was shown to be substantially increased in cells where bestrophin‐1 was deleted [37] consistent with an effect of bestrophin‐1 on L‐type Ca^2+^‐channel activity. Later, it was demonstrated that mutated bestrophin‐1 differentially modified both the activity of the Ca_v_1.3 and the trafficking of these channels to the cell membrane through physical interaction with the β4 auxiliary subunit associated with the Ca^2+^ channel [40, 41, 42, 43].

In pulmonary artery smooth muscle cells, TMEM16A colocalizes with L‐type Ca^2+^ channels (Ca_v_1.2) and the IP_3_‐receptor [44]. This conclusion was based on coimmunoprecipitation experiments, confocal and superresolution microscopy in combination with the use of TMEM16A knockout mice. Although all three channels in this cluster were required for a normal contractile response to serotonin, it was concluded that Ca^2+^ released from an IP_3_‐sensitive pool was the main driver of contraction, while the TMEM16A and L‐type Ca^2+^ channels contribute by refilling the sarcoplasmic reticulum after release of Ca^2+^. The study documents that a simple interaction between L‐type Ca^2+^ channels and TMEM16A does far from provide a full picture of the role of TMEM16A for excitation‐contraction coupling in smooth muscle cells.

In cardiomyocytes, bestrophin‐1, ‐2 and ‐3 are present [45], with bestrophin‐3 having the most abundant expression at the mRNA level and with the Ca^2+^‐activated Cl^−^ current having a potential role in phase 1 repolarization in the cardiomyocyte action potential. The expression of bestrophin‐3 was confirmed in canine and human heart cardiomyocytes [32] and TMEM16A is present in the mouse, canine and human heart [32, 46]. Immunohistochemistry of canine and human cardiomyocytes demonstrates that bestrophin‐3 and TMEM16A colocalize [32]. However, the authors did not consider the functional consequence of this colocalization. It is of interest to confirm these findings and provide an understanding of the consequences of this colocalization.

Collectively, these findings suggest that both bestrophins and TMEM16A participate in multi‐protein signaling complexes that modulate Ca^2+^ entry and release.

## 3 Bestrophin and TMEM16A May Regulate Release of Calcium from ER

In bestrophin‐1 knockout mice, ATP‐induced Cl^−^ secretion from tracheal epithelium is reduced at low ATP concentrations. At higher ATP concentrations no functional difference between wild‐type and knockout mice is seen [47]. The interpretation of this finding is that perhaps bestrophin‐1 modulates Cl^−^ secretion rather than being the actual channel in the plasma membrane [48]. These authors further suggested that bestrophin‐1 may regulate other channels including TMEM16A through regulation of Ca^2+^ release from the endoplasmic reticulum of human bronchial epithelial cells and mouse retinal pigment epithelial cells where a large part of the bestrophin‐1 appears to be located [14, 15]. The presence of bestrophin‐1 in the endoplasmic reticulum was further suggested by staining of retinal pigment epithelial cells with a bestrophin‐1 antibody, which indicated that bestrophin‐1 was proximal to but not within the basal plasma membrane [16]. Consistent with this location, knockdown of bestrophin‐1 reduced the release of Ca^2+^ from the endoplasmic reticulum induced by the SERCA pump inhibitor thapsigargin [49], and it is suggested that bestrophin‐1 may act as a counterion for the transfer of Ca^2+^ over the endoplasmic reticulum membrane [14, 15] thereby contributing to the control of cytosolic Ca^2+^ transients. Evidence was also provided that the resting cytosolic Ca^2+^ concentration in retinal pigment epithelial cells from bestrophin‐1 knockout mice is higher than in cells from wildtype mice [15, 49], and Ca^2+^ signals are suppressed in retinal pigment cells in a mouse with a mutated bestrophin‐1 [50]. However, evidence is also provided—as discussed above—that bestrophin‐1 may be placed in the basolateral aspect of retinal pigment epithelial cells where it provides a Cl^−^ conductance alongside TMEM16A [49, 51]. There is no information on whether the two Cl^−^ conductances have different roles or whether they supplement each other.

Interestingly, it has been suggested that also TMEM16A may control Ca^2+^ release from the endoplasmic reticulum [52, 53, 54]. As for bestrophin, TMEM16A may be close to the endoplasmic reticulum membrane and TMEM16A appears to tether the IP_3_‐receptor to the cell membrane [55]. Finally, knockout of TMEM16A in intestinal epithelial cells led to a reduction of carbachol‐induced Ca^2+^ transients [56].

In conclusion, research supports that bestrophins and TMEM16A may both be associated with IP_3_ receptors and that both can contribute to control of Ca^2+^ release from the endoplasmic reticulum. However, there is no direct evidence to demonstrate interaction between bestrophins and TMEM16A with respect to these functions.

## 4 Role for Bestrophin and TMEM16A in Pain Perception

Pain perception critically depends on depolarizing cation conductances, including the acid‐sensing ion channel 3 (ASIC3) and the transient receptor potential vanilloid 1 (TRPV1) channel expressed in dorsal root ganglion (DRG) neurons [57, 58]. In addition to cation influx, Cl^−^ conductance also contributes to pain signaling. In DRG neurons, intracellular Cl^−^ accumulates via the Na,K,2Cl co‐transporter (NKCC1) [59]. Upon opening of Cl^−^ channels, Cl^−^ efflux occurs, resulting in membrane depolarization and exacerbation of pain responses. Although GABA_A_ receptors mediate the major Cl^−^ current in these neurons [59], Ca^2+^‐activated Cl^−^ channels, bestrophin‐1 and TMEM16A, have been shown to play important regulatory roles. These channels may be activated downstream of TRPV1‐mediated Ca^2+^ influx, thereby linking cation entry to secondary Cl^−^‐dependent depolarization mechanisms [60], for review, see [61]. Both bestrophin‐1 and TMEM16A are expressed in the spinal cord and dorsal root ganglia—key sites for pain processing [62]. In tactile allodynia induced by REM sleep deprivation, bestrophin‐1 expression is increased in dorsal root ganglion cells, although there is no change of expression of bestrophin‐1 in dorsal spinal cord cells [63]. In contrast, TMEM16A expression is unchanged in dorsal root ganglion cells, whereas in dorsal spinal cord cells TMEM16A expression is decreased. In the same study, it was shown that inhibition of both bestrophin‐1 and TMEM16A provides an antiallodynic effect. Therefore, although both proteins are important for transfer of pain sensation to the brain, they are differentially regulated and may work through different mechanisms.

In conclusion, bestrophin and TMEM16A are important for processing pain, but the underlying mechanisms and functional consequences of their differential regulation remain unclear.

## 5 Role for Bestrophin and TMEM16A in Inflammation

It is well documented that TMEM16A plays an important role in inflammation [64]. Interestingly, the role of TMEM16A in inflammation is clearly reflected in the history of TMEM16A discovery. Before being defined as a Cl^−^ channel in 2008, TMEM16A was used as a marker for the severity of gastrointestinal cancers [65], and one of the important papers defining the role of TMEM16A as a Cl^−^ channel was based on enhancement of Ca^2+^‐ dependent Cl^−^ conductance with the proinflammatory interleukin 4 [18]. There is ample evidence from multiple tissues that TMEM16A plays an important role in inflammation: TMEM16A is important for the production of many proinflammatory signals, notably NF‐κB, several interleukins, ERK1/2, and Ca^2+^. Interestingly, the expression of TMEM16A is also regulated by several inflammatory factors, including several interleukins, histamine, and bradykinin. TMEM16A is thus part of a positive feedback loop in relation to inflammation (see Graphical Abstract). For a detailed description of the role of TMEM16A in inflammation see [64]. In airway differentiated cells, knockdown of TMEM16A reduced cytokine release, and the antiparasitic drug niclosamide reduced inflammation in the airway cells and inhibits TMEM16A, leading to the suggestion that part of the anti‐inflammatory response to niclosamide is caused by the effect on TMEM16A [54].

Interestingly, bestrophin‐1 has also been shown to have predictive power in relation to inflammation, for example, in cholangiocarcinoma [66], while inhibition of bestrophin‐1 function may lead to enhanced inflammation in the retina [67]. Furthermore, induction of inflammation with lipopolysaccharide (LPS), leads to enhanced expression of bestrophin‐1 mRNA and protein in microglia cells [68], and in cultured renal collecting duct cells LPS causes a transient increase of bestrophin‐1 protein [69]. In conclusion, there is a relationship between bestrophin and inflammation, albeit a complex one.

This complexity is also apparent in colitis. In the distal colonic epithelium, both bestrophin‐2 and TMEM16A are expressed, and in chemically induced colitis the transcripts of both bestrophin‐2 and TMEM16A are decreased. However, only TMEM16A is reduced at the protein level while bestrophin‐2 protein is unchanged [70]. The background for this complex regulation and its consequences remains unknown.

Vascular inflammation also seems to involve bestrophins and TMEM16A. The cells in the blood vessel wall can produce multiple proinflammatory mediators. Although there is no systematic assessment of the role of bestrophin and TMEM16A in vascular inflammation, there are numerous papers which strongly suggest that they do play a role.

Endothelial cells, smooth muscle cells and macrophages of the vascular wall express bestrophin‐3. The expression of bestrophin‐1 and bestrophin‐2 in the vascular wall is relatively lower, but there is evidence for their presence [2, 71]. Bestrophin‐3 is an important regulator of the inflammatory responses of cultured endothelial cells [72]. Up‐ and down‐regulation of bestrophin‐3 in human umbilical vein endothelial cells reveals that bestrophin‐3 inhibits TNFα‐induced NF‐κB activation, and this leads to the inhibition of release of cell adhesion molecules by the endothelial cells in vitro. These findings were further substantiated in vivo by showing that adenovirus infection with bestrophin‐3 protects against TNFα‐induced inflammation [72]. Furthermore, TNFα‐induced inflammation leads to decreased expression of bestrophin‐3 in endothelial cells both in vitro and in vivo [72]. Thus, there is a reciprocal interaction between bestrophin‐3 and TNFα‐induced inflammation.

Consistent with a “protective” role of bestrophin‐3 in vascular inflammation mediated via the endothelium, overexpression of bestrophin‐3 in vascular smooth muscle cells increases cell viability and prevents apoptosis, while downregulation of bestrophin‐3 potentiates H_2_O_2_‐induced apoptosis and decreases cell viability [73].

Macrophages are present in the healthy vascular wall: in an atherosclerotic blood vessel, the macrophages are increased in number, modified, and contribute to the development of atherosclerotic plaques. Macrophages express bestrophin‐1, and when the transcriptome of macrophages from the atherosclerotic plaque is compared with the transcriptome from normal tissue resident macrophages, a number of differentially expressed genes are found [74]. Principal component analysis followed by protein–protein interaction analysis points to bestrophin‐1 as being important, and the authors conclude that bestrophin‐1 is likely to be involved in the progression of inflammation in the atherosclerotic blood vessel [74].

TMEM16A is also associated with inflammatory changes in the vascular wall. This association is apparent from the relationship between hypertension, immune modulation and TMEM16A. Hypertension is associated with low grade chronic inflammation of the arteries, which is important for the remodeling of small arteries in hypertension [75]. Angiotensin II, which is a known proinflammatory substance, also induces low grade inflammation in small arteries [75]. Finally, the immune system is known to have several diverse roles in blood pressure regulation [76]. It is therefore of interest that TMEM16A expression is increased and seems to play a role for the vascular pathology in mesenteric [77] and coronary [78] arteries from spontaneously hypertensive rats and in aorta from mice with hypertension induced by a high NaCl diet combined with L‐arginine methyl ester (L‐NAME) treatment [79].

Knockout of TMEM16A in smooth muscle cells and pericytes reduces blood pressure and the blood pressure increase to angiotensin II [80]. These effects on blood pressure are mediated by a decrease in peripheral resistance and are consistent with omission of the depolarizing effect of the Ca^2+^‐activated Cl^−^ current in smooth muscle cells and pericytes [80]. However, other antihypertensive effects of knocking out TMEM16A cannot be excluded.

Endothelial cell‐specific knockout of TMEM16A also lowers blood pressure. This effect is dependent on TMEM16A‐induced stabilization of the ROS producing NADPH oxidase activity in endothelial cells [81]. This role of TMEM16A in endothelial function translates to blood pressure and the blood pressure increase by angiotensin II infusion, which is blunted in arteries from endothelial cell‐specific TMEM16A knockout mice [81].

A specific role for TMEM16A for vascular inflammation was addressed by Zeng and colleagues [79]. These authors set out from the observation that Arctigenin reduces blood pressure and superoxide anion production in arteries from spontaneously hypertensive rats [82]. Arctigenin is a bioactive constituent from dried seeds of *Arctium lappa* used in traditional Chinese medicine. In mice with a high NaCl/L‐NAME induced hypertension, the expression of TMEM16A and the inflammatory markers VCAM‐1 and ICAM‐1 were increased [82]. Treatment with Arctigenin reduced blood pressure (as in the spontaneously hypertensive rats), and importantly, also reduced TMEM16A, VCAM‐1, and ICAM‐1 expression. These effects were confirmed in mouse aortic smooth muscle cells cultured in high (169 mM) NaCl medium leading to a high TMEM16A, VCAM‐1, and ICAM‐1 expression. The authors [79] provided further evidence that two genes downstream of TMEM16A—endothelial cell‐specific molecule 1 (ESM1) and CXC chemokine ligand 16 (CXCL16)—were important for the effect of TMEM16A on VCAM‐1 and ICAM‐1. This study thus provides direct evidence for the role of TMEM16A in blood pressure regulation and vascular inflammation. It will be important to understand to what extent the effects of TMEM16A on blood pressure and inflammation are causally related.

Experimentally induced pulmonary hypertension is also associated with upregulation of the Ca^2+^‐activated Cl^−^ current and TMEM16A expression in pulmonary artery smooth muscle cells [83, 84]. Accordingly, in patients with idiopathic pulmonary hypertension, the Ca^2+^‐activated Cl^−^ current and TMEM16A expression are strongly upregulated in pulmonary artery smooth muscle cells [85]. The mechanism(s) responsible for the upregulation of TMEM16A was not addressed in these studies. Furthermore, in two models of pulmonary hypertension, treatment with the TMEM16A inhibitor benzbromarone reduced pulmonary pressure and limited vascular remodeling, while silencing TMEM16A expression reversed the enhanced depolarization and reduced proliferation of pulmonary artery smooth muscle cells from patients with pulmonary hypertension [85]. The mechanism involved in upregulation of TMEM16A in pulmonary hypertension may relate to the associated pulmonary inflammation or to the effect of endothelin, which is increased in patients with pulmonary hypertension [86]. Importantly the endothelin‐induced upregulation of TMEM16A seen in these studies was prevented by applying the anti‐inflammatory lipids docosahexaenoic acid monoacylglyceride (MAG‐DHA) or Resolvin D1 [86]. Another possible mediator of the regulation of TMEM16A expression is hypoxia, which has been shown to increase the Ca^2+^‐activated Cl^−^ current (most likely mediated by TMEM16A) in mouse coronary endothelial cells [87]. The evidence for the presence of TMEM16A and its functional role in pulmonary arteries in pulmonary hypertension is strong and a role for the associated vascular inflammation may be important.

In contrast to the increased expression of TMEM16A in hypertension, the basilar artery smooth muscle cells from renal hypertensive rats (2‐kidney, 2 clip hypertension) had reduced expression of the Ca^2+^‐activated Cl^−^ conductance and decreased expression of TMEM16A [71]. This is counter to the prevailing idea that inflammation is associated with an increased expression of TMEM16A. However, while the authors recognized this, they did not discuss it. Furthermore, angiotensin II induced proliferation of smooth muscle cells from the rat basilar artery was facilitated by knockdown of TMEM16A and inhibited by overexpression of TMEM16A [71]. This effect of TMEM16A occurs through inhibition of cyclin D1 and cyclin E expression and consequent arrest of the cell cycle in G0/G1 phase. It is of substantial interest to understand whether these effects are mediated via the role of TMEM16A as a Cl^−^ channel or whether it is mediated through an unrelated effect of TMEM16A. It will also be important to elucidate whether this discrepancy in the role of TMEM16A in vascular remodeling and contractility reflects differences between vascular beds (i.e., pulmonary vs. cerebral arteries) or is a consequence of experimental conditions. Altogether, both TMEM16A and bestrophins are implicated in inflammatory processes; but whether any interaction between them is important for the inflammatory response remains unknown.

In conclusion, bestrophins and TMEM16A are widely co‐expressed and often influence overlapping physiological processes. In vascular smooth muscle, evidence supports functional cooperation, colocalization, and regulatory crosstalk. However, the molecular basis of this interaction remains unresolved. Surprisingly little is known about the role of Cl^−^ conductance per se, or of intracellular Cl^−^ concentration, in the diverse functions of bestrophins and TMEM16A. This is particularly true for the many transcriptional and translational effects shown for TMEM16A (see Matchkov et al. [33]). Future studies should clarify whether the transcriptional and translational effects attributed to TMEM16A—and potentially to bestrophins—are mediated by changes in intracellular Cl^−^ concentration, channel‐independent signaling functions, or protein–protein interactions.

## Author Contributions

**Holger Nilsson:** conceptualization, validation, writing – review and editing. **Christian Aalkjær:** writing – original draft, validation, writing – review and editing, conceptualization. **Donna B. Boedtkjer:** conceptualization, validation, writing – review and editing. **Vladimir Matchkov:** conceptualization, validation, writing – original draft, writing – review and editing. **Vibeke Secher Dam:** conceptualization, validation, writing – review and editing.

## Funding

This work was supported by Lundbeck Foundation (R344‐2020‐952, R412‐2022‐449) and Danmarks Frie Forskningsfond (3101‐00103B).

## Disclosure

There is no previously published figure in this review.

## Ethics Statement

The authors have permission to perform animal experiments. There is no human data discussed in this review.

## Conflicts of Interest

The authors declare no conflicts of interest.

## Data Availability

Data sharing not applicable to this article as no datasets were generated or analysed during the current study.
